# 3, 4-dihydroxyl-phenyl lactic acid restores NADH dehydrogenase 1 α subunit 10 to ameliorate cardiac reperfusion injury

**DOI:** 10.1038/srep10739

**Published:** 2015-06-01

**Authors:** Xiao-Yuan Yang, Ke He, Chun-Shui Pan, Quan Li, Yu-Ying Liu, Li Yan, Xiao-Hong Wei, Bai-He Hu, Xin Chang, Xiao-Wei Mao, Dan-Dan Huang, Li-Jun Wang, Shui-Wang Hu, Yong Jiang, Guo-Cheng Wang, Jing-Yu Fan, Tai-Ping Fan, Jing-Yan Han

**Affiliations:** 1 Tasly Microcirculation Research Center, Peking University Health Science Center, Beijing, China; 2 Department of Integration of Traditional Chinese and Western Medicine, School of Basic Medical Sciences, Peking University, Beijing, China; 3 Department of Biophysics, Peking University Health Science Center, Beijing, China; 4 Department of Pathophysiology and Key Laboratory of Proteomics of Guangdong Province, Southern Medical University, Guangzhou, China; 5 Angiogenesis & Chinese Medicine Laboratory, Department of Pharmacology, University of Cambridge, UK; 6 Key Laboratory of Microcirculation, State Administration of Traditional Chinese Medicine of the People’s Republic of China; 7 Key Laboratory of Stasis and Phlegm, State Administration of Traditional Chinese Medicine of the People’s Republic of China

## Abstract

The present study aimed to detect the role of 3, 4-dihydroxyl-phenyl lactic acid (DLA) during ischemia/reperfusion (I/R) induced myocardial injury with emphasis on the underlying mechanism of DLA antioxidant. Male Spragu-Dawley (SD) rats were subjected to left descending artery occlusion followed by reperfusion. Treatment with DLA ameliorated myocardial structure and function disorder, blunted the impairment of Complex I activity and mitochondrial function after I/R. The results of 2-D fluorescence difference gel electrophoresis revealed that DLA prevented the decrease in NDUFA10 expression, one of the subunits of Complex I. To find the target of DLA, the binding affinity of Sirtuin 1 (SIRT1) to DLA and DLA derivatives with replaced two phenolic hydroxyls was detected using surface plasmon resonance and bilayer interferometry. The results showed that DLA could activate SIRT1 after I/R probably by binding to this protein, depending on phenolic hydroxyl. Moreover, the importance of SIRT1 to DLA effectiveness was confirmed through siRNA transfection *in vitro*. These results demonstrated that DLA was able to prevent I/R induced decrease in NDUFA10 expression, improve Complex I activity and mitochondrial function, eventually attenuate cardiac structure and function injury after I/R, which was possibly related to its ability of binding to and activating SIRT1.

Coronary heart disease is a leading cause of death all over the world, and its incidence is increasing at an alarming rate^1^. After an acute myocardial infarction, percutaneous coronary intervention has become the most common strategy to restore myocardial perfusion, however it does not reduce the risk of mortality^2^, due to numerous complications of I/R injury. I/R causes cardiomyocyte death, myocardial stunning and left ventricular remodeling, which contribute to the reduction of cardiac output and myocardial fibrosis leading to the development of heart failure^3^,^4^.

One of the major consequences of I/R is mitochondrial dysfunction, resulting in mitochondria-derived myocardium damage by reactive oxygen species (ROS) production, adenosine triphosphate (ATP) reduction, increased mitochondrial permeability, cytochrome *c* release and activation of programmed cell death pathways^5^. The mitochondrial electron transport chain Complex I and Complex III are the two sites that generate approximately 90% of cellular ROS, where electrons escape from the electron transport chain (ETC), react with molecular oxygen and generate superoxide^6^. Complex I activity markedly reduces during I/R due to a decrease in the reduced nicotinamide adenine dinucleotide (NADH) dehydrogenase component^7^, which leads to augmented electron leakage and ROS generation^8^. Given to the central role of the mitochondria in I/R injury, preventing ETC (e.g., Complex I) subunit reduction and maintaining ETC enzyme activity are considered to be effective strategies in alleviating I/R induced injury.

NADH dehydrogenase (ubiquinone) 1 alpha subcomplex 10 (NDUFA10) belongs to accessory subunits of Complex I. Microarray analysis and proteomics results revealed that alterations in NDUFA10 protein or mRNA expression are associated with several diseases, including cardiac hypertrophy^9^, type 2 diabetes^10^ and Leigh disease^11^, etc. Hoefs *et al.*^11^ reported that patients with two mutations in NDUFA10 gene display a marked decrease in Complex I activity and a disturbed assembly or stability of Complex I. It is worth noticing that NDUFA10 may serve as a NAD(H) binding subunit^12^.

Recent studies suggest that changes in Sirtuin1 (SIRT1) protein content, a nicotinamide adenine dinucleotide (NAD^+^) dependent deacetylases, result in alternations in mitochondrial genes expression and enzymes activity^13^,^14^. SIRT1 affects a wide range of physiological and pathophysiological processes, including metabolism, cell survival, cancer, aging and calorie restriction^15^,^16^. One of the most important roles of SIRT1 is to modulate the expression of mitochondrial genes and proteins. It was reported that SIRT1 induces expression of mitochondrial respiration chain genes, including cytochrome c oxidase subunit Va (COXVa) and cytochrome *c*, through deacetylation of PGC-1α^17^. Resveratrol, a potent SIRT1 activator, was reported to enhance the gene expression of components of respiratory chain (e.g., NADH dehydrogenase [ubiquinone] 1 beta subcomplex 8) and oxidative phosphorylation enzymes (e.g., COXVa)^18^. However, whether SIRT1 could modulate NDUFA10 expression remains unclear. Accumulating evidence proves that SIRT1 plays an important role in preventing cardiovascular diseases and regulating myocardium survival^16^. Thus, pharmacological activation of SIRT1 might be an efficient strategy to prevent hearts from I/R injury.

3, 4-dihydroxyl-phenyl lactic acid (DLA) is a major ingredient of cardiotonic pills® (CP), a Traditional Chinese Medicine that has been scheduled to undergo phase III clinical trials for treatment of ischemic cardiovascular diseases by the US Food and Drug Administration in 2013. Our previous results indicated that CP ameliorates I/R-induced myocardial damage and fibrosis in rats^19^,^20^. DLA itself is well known for its cardiovascular protective effects including coronary vasodilatation^21^, antioxidant activity^22^,^23^, and reduction in endothelial permeability^24^. It was found that the combined use of puerarin and DLA significantly reduces acute ischemic myocardial injury^25^. In addition, DLA could scavenge superoxide anion induced by I/R^26^. However, whether the major consequences of I/R mitochondrial dysfunction and ETC subunit reduction could be regulated by DLA is still unknown, and the intracellular target for the beneficial actions of DLA is so far unclear. Thus, in the present study we tested whether SIRT1 turns out to be regulated by DLA to increase NDUFA10 expression and Complex I enzyme activity, and ultimately reduce ROS generation and I/R-induced myocardial injury.

## Results

### DLA administration diminishes infarct size, preserves myocardium structure and reduces leukocyte infiltration after myocardial I/R

**Figure 1:**
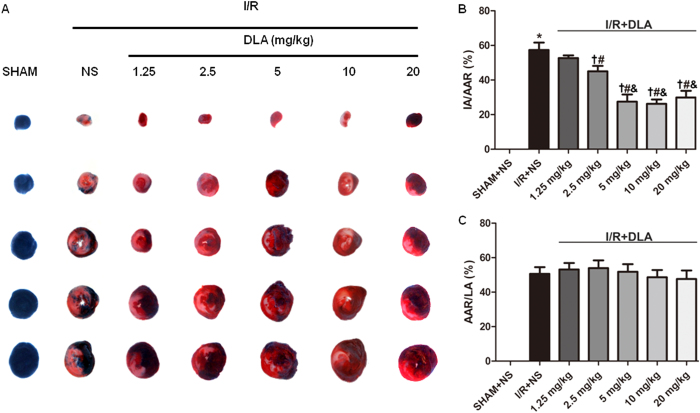
DLA administration diminishes I/R-induced infarct. **A**, Representative images of myocardial tissue sections stained with TTC and Evans blue. The rats in I/R groups were subjected to 30 min ischemia and 90 min reperfusion with intravenous injection of normal saline (NS) or DLA (dose ranging from 1.25 mg/kg to 20 mg/kg). The non-ischemic area is indicated by blue, the AAR by red and the IA by white. **B** and **C**, Quantitative analysis of infarct size of myocardial tissues. The ratios of IA to AAR and AAR to LA are shown. Results are presented as mean ± S.E.M (n = 6) * *p* < 0.05 *vs.* SHAM+NS group, # *p* < 0.05 *vs.* I/R+NS group, † *p* < 0.05 *vs* I/R+DLA 1.25 mg/kg, & *p*<0.05 *vs* I/R+DLA 2.5 mg/kg.

We first examined whether DLA exhibited a cardioprotective role during I/R in rats. Upon I/R, the hearts from DLA treated rats showed a reduced ratio of IA/AAR in a dose-dependent manner compared to those from saline treated rats (Fig. 1A,B). However, the ratios of AAR/LA were the same in all I/R groups regardless of DLA treatment or not, indicating a similar tension and placement of the ligature among the groups (Fig. 1C). The dose of 5 mg/kg was selected as an optimal dose of DLA and applied in all subsequent experiments.

**Figure 2:**
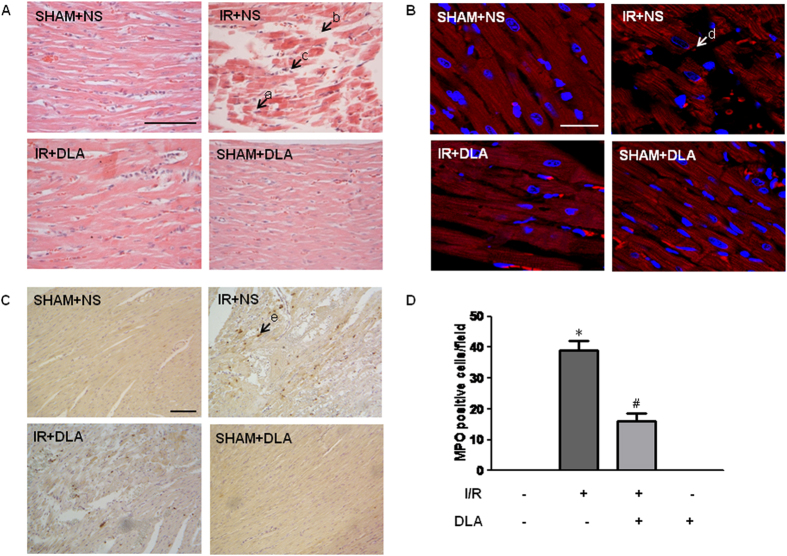
DLA administration attenuates I/R-induced morphological alterations and leukocyte infiltration. **A**, Representative photographs of myocardial tissues stained with hematoxylin and eosin. Rats were subjected to sham operation and administrated with normal saline (SHAM+NS) or DLA (5 mg/kg, SHAM+DLA), or to I/R with normal saline (I/R+NS) or DLA (5 mg/kg, I/R+DLA). Bar = 100 μm. a: disordered and disrupted myocardial fiber. b: interstitial edema. c: inflammatory cell infiltration. **B**, Representative myocardial tissue sections stained for F-actin and nuclei (red and blue, respectively, in overlays). F-actin is labeled by rhodamine phalloidin. Bar = 25 μm. d: disordered and disrupted myocardial F-actin. **C**, Photomicrographs of immunohistochemistry staining for myeloperoxidase (MPO) in myocardium of each group, respectively. Bar = 100 μm. e: MPO-positive cells in myocardium. **D**, Quantitative evaluation of MPO-positive cells infiltrating into myocardial tissue in different groups. Results are presented as mean ± S.E.M (n = 6) * *p* < 0.05 *vs.* SHAM+NS group, # *p* < 0.05 *vs.* I/R+NS group.

Micrographs of hematoxylin/eosin (H&E) staining sections shown in Fig. 2A revealed that myocardial tissues from rats undergoing I/R were morphologically altered, including disordered arrangement and disruption of myocardial fibers, myocardial interstitial edema and inflammatory cell infiltration. Noticeably, administration of DLA markedly protected hearts from morphological alterations after I/R. F-actin, as a component of thin filament, has an important role in contraction force generation^27^. Confocal microscopy studies showed a pronounced F-actin disarrangement and disruption in myocardium sections after challenge by I/R which were attenuated by DLA treatment (Fig. 2B).

As a marker enzyme of neutrophils, MPO expression in myocardium was assessed by immunohistochemistry. Few cells exhibited MPO-positive staining in SHAM group. On the contrary, I/R evoked a considerable increase in infiltration of neutrophils. Interestingly, the I/R-induced increase in the number of MPO-positive cells in heart was diminished by treatment with DLA (Fig. 2C). The quantitative result of the MPO-positive cells in surrounding infarction areas in myocardium is consistent with the qualitative survey, as shown in Fig. 2D.

### DLA administration leads to better preservation of LV function and MBF after I/R

**Figure 3:**
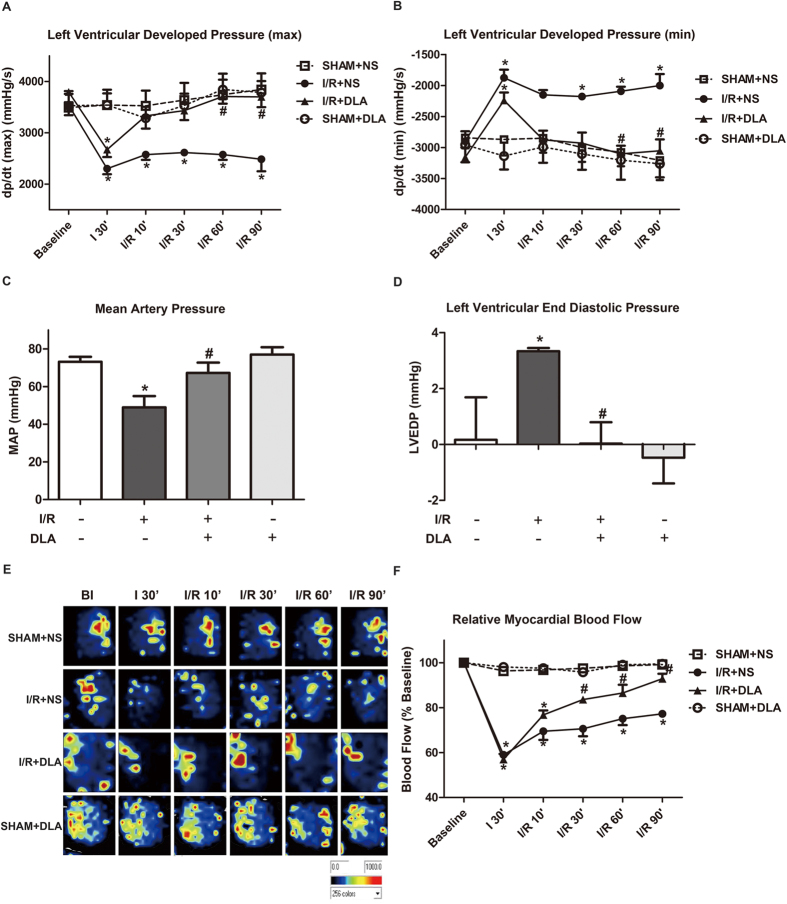
DLA administration results in better recovery of LV function and MBF. **A** and B, Results of left ventricular developed pressure (max) and left ventricular developed pressure (min) before ischemia (baseline), after 30 min of ischemia (I 30’), and after 10 min (I/R 10’), 30 min (I/R 30’), 60 min (I/R 60’) and 90 min of reperfusion (I/R 90’) in four groups, respectively. **C** and **D**, Effect of DLA administration on MAP and LVEDP at the end of reperfusion in various groups. **E**, Representative MBF pictures acquired by Laser Doppler Perfusion Imager at different time points in 4 different groups. Color scale illustrates MBF with dark blue through red representing low to high flow. **F**, Statistical results of MBF. MBF is expressed as a percentage of baseline MBF. Three images were acquired and evaluated for each rats at each time point. Results are presented as mean ± S.E.M (n = 6) * *p* < 0.05 *vs.* SHAM+NS group, # *p* < 0.05 *vs.* I/R+NS group.

Given to the fundamental role of F-actin in generating contractile force, we then tested whether DLA treatment would benefit cardiac function during I/R. LV function at baseline was comparable among four groups. LV developed pressure (max) fell substantially at 30 min of ischemia compared to sham operation. However, DLA treatment improved LV developed pressure (max) considerably compared with saline treated group during reperfusion (Fig. 3A). Similar results were observed for LV systolic pressure (Supplementary Fig. S1A). Furthermore, reduction in mean artery pressure (Fig. 3C) and artery systolic pressure (Supplementary Fig. S1B) induced by I/R was notably restored by DLA administration after 90 min of reperfusion. In addition, LV developed pressure (min) rose dramatically at 30 min of ischemia compared with SHAM group, DLA treated rats exhibited better recovery of LV developed pressure (min) than normal saline treated group during reperfusion (Fig. 3B). Consistent with the results of LV developed pressure (min), increase in LV end diastolic pressure (Fig. 3D) caused by I/R was substantially prevented by DLA administration at the end of reperfusion. Taken together, these results demonstrated that the impaired LV function in response to I/R was significantly protected in DLA treated group. The protective role of DLA for heart structure and function was further confirmed by echocardiography analysis conducted at the third week after I/R. As shown in Supplementary Table S3 and Fig. S4, compared with SHAM group, I/R led to an obvious increase in LVIDd, LVIDs, LV Vold and LV Vols, but a decrease in EF%, FS% and FAC%, most of which, except LVIDd and LV Vold, were attenuated by DLA treatment.

Better recovery of left ventricular function suggested that DLA treatment might improve coronary perfusion after I/R. To verify this speculation we then examined MBF by using a Laser Doppler Perfusion Imager. Representative Laser Doppler pictures of MBF at different time points of I/R were acquired from four groups, as illustrated in Fig. 3E. The time courses of quantitative results showed that MBF fell evidently after 30 min of ischemia compared to SHAM+NS groups, however, DLA treatment led to a better MBF recovery in comparison with I/R+NS group (Fig. 3F).

### DLA administration represses I/R-induced apoptosis

TUNEL staining was performed to investigate the extent of apoptosis in the AAR region in four groups. Confocal images and statistical results of TUNEL staining indicated that the hearts from DLA treated rats showed a significant decrease in the number of TUNEL positive cells compared to that from I/R+NS group after I/R, whereas only few TUNEL positive cells were detected in the hearts from SHAM group (Supplementary Fig. S2A and B). Apoptosis is a process that is controlled by apoptosis related proteins, thus we assessed Bcl-2, Bax and Cleaved-caspase 3 in whole-heart lysates. The results demonstrated that DLA exerted anti-apoptosis role by upregulating the expression of anti-apoptotic protein Bcl-2 and downregulating the expression of pro-apoptotic protein Bax, as compared to rats in I/R+NS group (Supplementary Fig. S2C and D). Furthermore, ratio of Bcl-2/Bax, a determinant of the susceptibility of myocardium to apoptosis, was noticeably diminished after I/R, which was restored in the presence of DLA (Supplementary Fig. S1C). Consistent with these results, DLA prevented hearts from I/R induced increase in caspase-3 cleavage (Supplementary Fig. S2E).

### DLA administration restores I/R induced reduction in NDUFA10 expression

**Figure 4:**
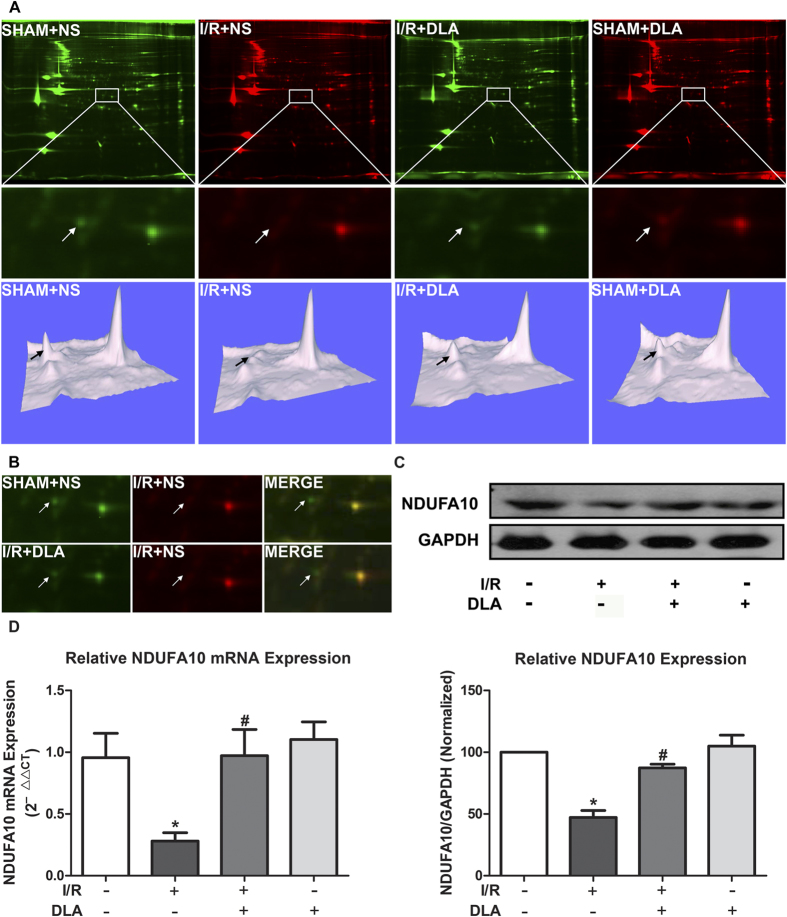
DLA administration restores I/R induced reduction in NDUFA10 expression. **A**, Representative 2D-DIGE images of heart lysates from ischemic region in various groups. Selected and enlarged regions of 2D-DIGE images displaying NDUFA10 are shown in the middle panel, while three-dimensional views of the selected regions displaying NDUFA10 expression alterations shown in the lower panel. Arrows indicate NDUFA10. **B**, The overlay images with yellow spots indicating proteins that have equal expression levels in both groups, green spots indicating proteins down-regulated in I/R+NS group. Arrows indicate NDUFA10. **C**, Western blot and statistical analysis of NDUFA10 based on the data of 3 independent experiments normalized to GAPDH. The gels have been run under the same experimental conditions. **D**, NDUFA10 mRNA levels determined by reverse transcription real-time PCR. Quantitative results of mRNA expression were derived from 5 independent experiments and normalized to GAPDH. Results are presented as mean ± S.E.M. * *p* < 0.05 *vs.* SHAM+NS group, # *p* < 0.05 *vs.* I/R+NS group.

2-D fluorescence difference gel electrophoresis (2D-DIGE) was performed in order to reveal the mechanism by which DLA protects hearts from I/R injury. Representative 2D gel images of all groups are shown in Fig. 4A. Four protein spots were identified as differentially expressed between I/R+NS group and I/R+DLA group (Supplementary Table S1). Among the identified proteins, NDUFA10 was the only protein located in mitochondria. Mitochondria is the major contributor to the oxidative damage and myocardium apoptosis under the conditions of I/R^7^. Considering the role of DLA in antioxidation and anti-apoptosis, we focused on the study of mitochondrial protein NDUFA10.

Selected regions of the 2D gels displaying NDUFA10 protein spot and its three-dimension view in Fig. 4A and B showed that I/R induced a dramatic reduction in NDUFA10 expression in comparison with sham operation, and this reduction was protected by treatment of DLA. Western blot confirmed the results of 2D-DIGE (Fig. 4C). NDUFA10 mRNA levels were also assessed by reverse transcription real-time PCR. Likewise, DLA administration dramatically restored the reduction in NDUFA10 mRNA expression induced by I/R (Fig. 4D).

### DLA administration prevents against the impairment in Complex I function induced by I/R

**Figure 5:**
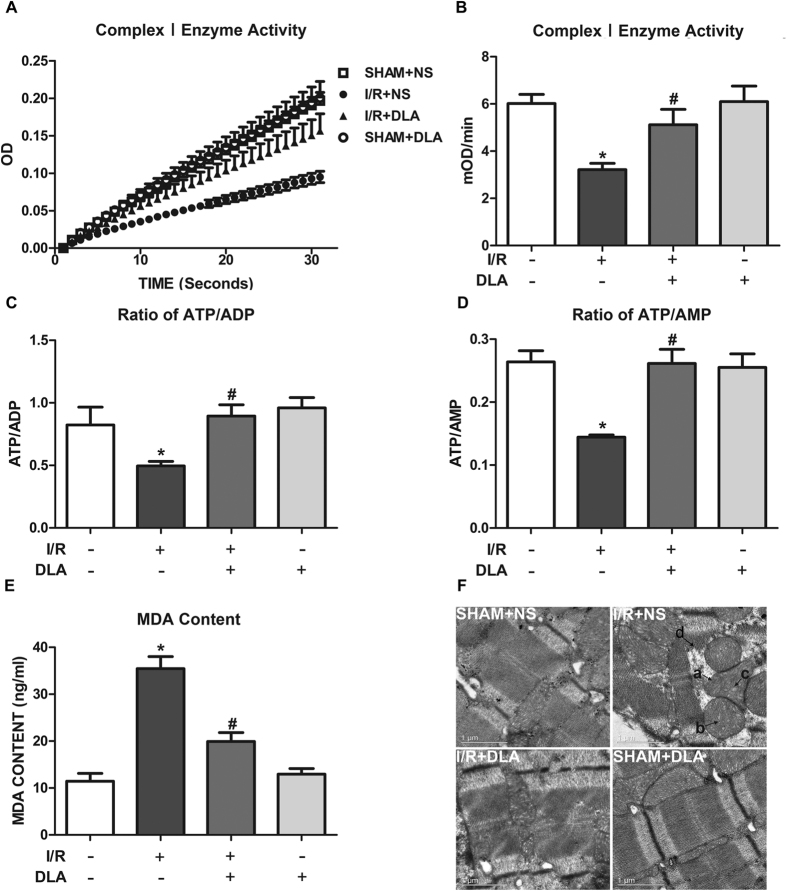
DLA administration restores Complex I activity and mitochondrial function after I/R. **A**, Time course of OD at 450 nm, the slope being proportional to Complex I activity. **B**, Complex I activity based on the slope of increase in OD at 450 nm. **C** and **D**, Quantitative results of ratios of ATP/ADP and ATP/AMP in ischemic heart homogenates in various groups after I/R, respectively. **E**, Statistical analyses of MDA of ischemic heart homogenates in various groups after I/R. **F**, Representative electron micrographs of myocardial tissues of rats from 4 groups. a: mitochondrial swelling. b: disarranged and obscure crista. c: vacuoles among crista. d: disrupted myofilament and sarcomere. Results are presented as mean ± S.E.M (n = 6) * *p* < 0.05 *vs.* SHAM+NS group, # *p* < 0.05 *vs.* I/R+NS group.

Given to the fact that NDUFA10 is essential for proper function of Complex I^11^,^28^, we speculated that NDUFA10 decrement would result in dysfunction of Complex I and mitochondria in the heart. To test this hypothesis, mitochondrial Complex I activity was first determined, and the results are shown in Fig. 5A in which OD at 450 nm increased over time with the slope being proportional to Complex I activity. Evidently, impaired Complex I activity induced by I/R was remarkably reversed by injection of DLA (Fig. 5B).

Increased Complex I activity was expected to improve mitochondrial respiration, resulting in enhanced ATP production and less free radical accumulation. Therefore, energy metabolic conditions and redox state of myocardial tissue were next assessed. Consistent with the preserved Complex I activity, the reduction in myocardial ratio of ATP/ADP and ATP/AMP after I/R was considerably restored by DLA administration (Fig. 5C,D). In addition, I/R induced an increase in the level of malonidialdehyde (MDA), an indicator of cellular lipid peroxidation, which was dramatically reduced by administration of DLA (Fig. 5E). A decrease in Complex I activity would impair the mitochondrion themselves. Mitochondrial ultrastructure observed by transmission electron microscopy (Fig. 5F) showed that I/R resulted in distinctive ultrastructure alterations, including disordered mitochondrion distribution with disarranged and obscure crista and vacuoles within the matrix, accompanying by disrupted myofilament and sarcomere. Noticeably, these deleterious effects of IR were markedly attenuated by DLA administration (Fig. 5F).

### DLA binds to and activates SIRT1 to preserve NDUFA10 expression and Complex I activity

**Figure 6:**
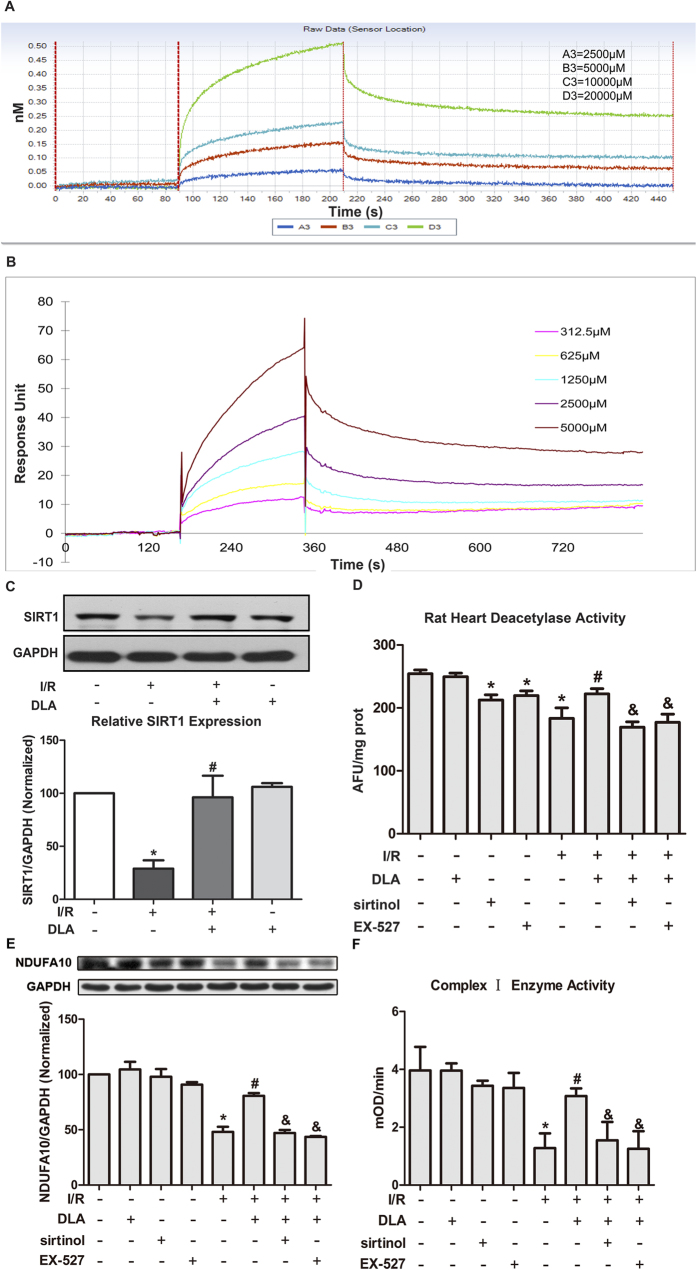
DLA binds to and activates SIRT1. **A**, Sensorgram binding curves of four different concentrations of DLA (2500, 5000, 10000, and 20000 μM, curves from bottom to top) measured by BLI. **B**, Representative sensorgrams obtained from the injections of DLA at concentrations of 312.5, 625, 1250, 2500, and 5000 μM (curves from bottom to top) using SPR. **C**, Representative western blot bands of SIRT1 in various groups shown above and quantitative results below. The gels have been run under the same experimental conditions. **D**, The deacetylase activity of rats myocardial tissue (n = 6) in various groups with or without SIRT1 inhibitor sirtinol and EX-527. **E**, The representative western blot bands and the quantitative results of NDUFA10 in various groups with or without sirtinol and EX-527. The gels have been run under the same experimental conditions. Quantification results for NDUFA10 band intensities were normalized to GAPDH from 3 independent experiments. **F**, Complex I activity based on the slope of increase in OD at 450 nm in all groups with or without sirtinol and EX-527. Results are presented as mean ± S.E.M. * *p* < 0.05 *vs.* SHAM+NS group, # *p* < 0.05 *vs.* I/R+NS group, & *p* < 0.05 *vs.* I/R+DLA group.

The question arose was that what was the target upstream NDUFA10 for DLA acting at? To this end, SIRT1 has been reported to induce several mitochondrial respiration chain gene expression, it is thus likely that DLA elevates NDUFA10 mRNA levels through activating SIRT1. To test this hypothesis, BLI was conducted to determine the binding affinity of DLA to SIRT1. As shown in Fig. 6A, DLA was able to bind to SIRT1 in a dose-dependent manner. K_D_ derived from recorded BLI curve were 3.37 × 10^-4^ (M). To verify the results of BLI, the binding capacity of DLA to SIRT1 was analyzed by SPR assay. Consistent with BLI, results of SPR indicated that DLA bound to SIRT1 in a dose-dependent way (Fig. 6B). K_D_ constant of DLA binding to SIRT1 was 7.84 × 10^−4^ (M).

DLA having a capacity of directly interacting with SIRT1 led us to ask: what is the impact of DLA on SIRT1 content and activity? As shown in Fig. 6C, DLA protected hearts from I/R-caused reduction in SIRT1 protein expression. SIRT1 inhibitors sirtinol and EX-527 were applied to determine the effects of DLA on deacetylase activity of SIRT1. Doses of inhibitors were calculated based on the concentration used *in vitro* experiments. Optimal inhibitor dose was selected from three doses administrated, the dose of sirtinol and EX-527 applied in subsequent experiments was 4.8 mg/kg and 4 mg/kg, respectively. Based on the results of *in vivo* deacetylase activity detection, impaired deacetylase activity induced by I/R was remarkably reversed by injection of DLA, while preserved deacetylase activity caused by DLA administration during I/R was abolished by SIRT1 inhibitor sirtinol and EX-527 (Fig. 6D). DLA treatment dramatically brought down increased acetylated-Foxo-1 (Ac-Foxo-1) levels after I/R (Supplementary Fig. S3A), which verified the results of SIRT1 activity. Moreover, manganese superoxide dismutase (MnSOD), which could be upregulated by SIRT1 in the transcriptional level^29^, was obviously decreased after I/R, which was reversed by DLA administration as well (Supplementary Fig. S3B).

Furthermore, the beneficial effects of DLA on restoring NDUFA10 expression (Fig. 6E) and Complex I activity (Fig. 6F) were abolished by sirtinol and EX-527. The result of present study implied NDUFA10 as a downstream effecter of SIRT1 activation by DLA administration.

### DLA binds to SIRT1 in a phenolic hydroxyl dependent manner

**Figure 7:**
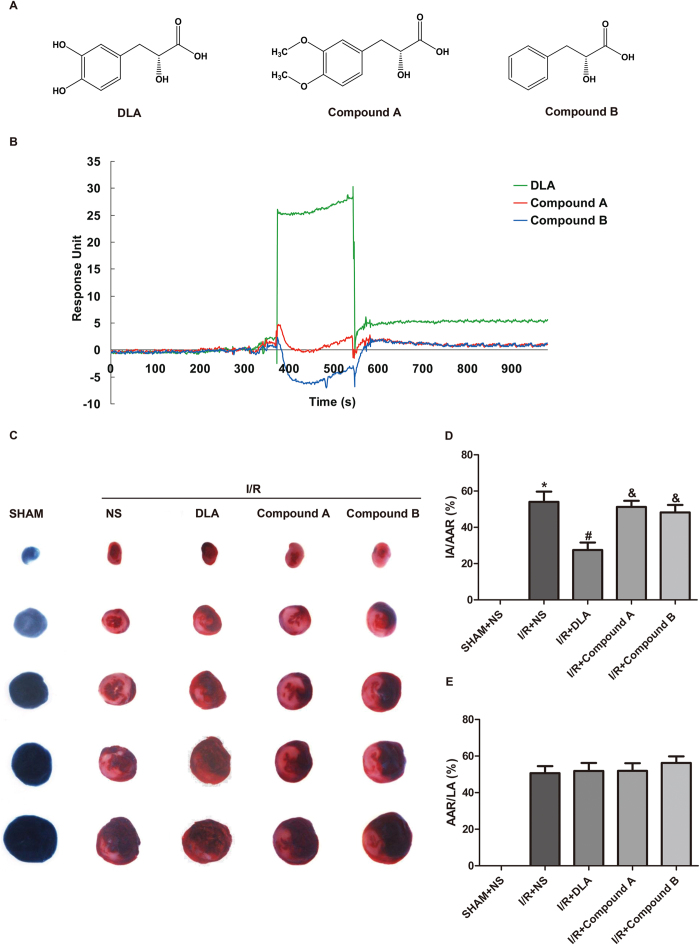
DLA derivatives exhibit lower affinity for SIRT1 and impaired ability for reducing infarct size after I/R. **A**, The structures of DLA, Compound A and Compound B. **B**, SPR binding curves of DLA, Compound A and Compound B to SIRT1, subtracting non-specific binding to bovine serum albumin (BSA), at the same concentration of 1250 μM. **C**, Representative images of myocardial tissues stained with TTC and Evans blue in rats of SHAM group and rats subjected to I/R with or without injection of DLA (5 mg/kg), Compound A (5 mg/kg) and Compound B (5 mg/kg). **D** to **E**, Quantitative analyses of infarct size in myocardial tissues. The ratios of IA to AAR (**D**), and AAR to LA (**E**) are shown. Results are presented as mean ± S.E.M (n = 6) * *p* < 0.05 *vs.* sham group, # *p* < 0.05 *vs.* I/R group, & *p* < 0.05 *vs.* I/R+DLA group.

Then we tried to elucidate the mechanisms of how DLA could bind to SIRT1. DLA derivatives were synthesized by replacing both phenolic hydroxyl groups of DLA into methoxyl (Compound A) or hydroxyl (Compound B) (Fig. 7A). The association constant (k_a_), dissociation constant (k_d_), and K_D_ of DLA, Compound A and B binding to SIRT1 were summarized in Supplementary Table S2, showing that the K_D_ constants of Compound A and B binding to SIRT1 were 0.638 (M) and 0.123 (M), respectively. K_D_ values and binding curves of three molecules to SIRT1 shown in Fig. 7B indicated that both Compound A and B exhibited lower affinity than DLA for SIRT1. Furthermore, the beneficial effects of DLA on preventing myocardial infarct during I/R was abolished by replacement of phenolic hydroxyl groups, Compound A and B exhibiting an impaired ability to decrease IR-caused myocardial infarct compared with DLA (Fig. 7C–E). Taken together, these results demonstrated that cardioprotective effect of DLA during I/R was correlated with its binding to and activating SIRT1, a process depending on the presence of phenolic hydroxyl groups.

### SIRT1 is required for the protective role of DLA in increasing NDUFA10 expression and Complex I activity, reducing mitochondrial ROS and cell death

**Figure 8:**
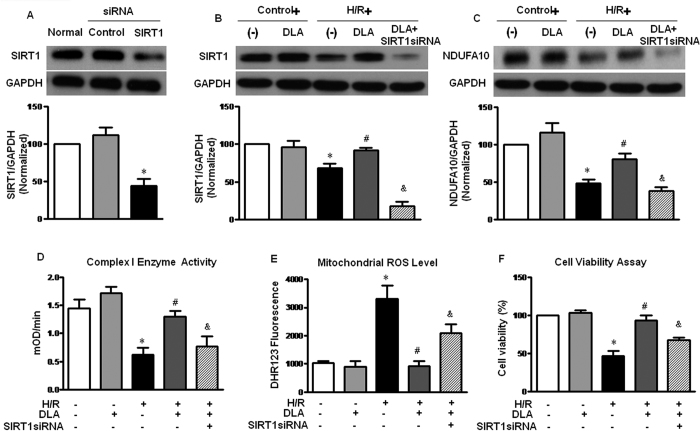
Knockdown of SIRT1 in H9c2 cells reduces the protective role of DLA after H/R. **A**, Western blot bands and statistical analysis of SIRT1 in H9c2 cells treated with control siRNA or SIRT1 siRNA. The quantification was based on the data of 4 independent experiments and normalized to GAPDH. **B** and **C**, Representative western blot bands of SIRT1 and NDUFA10 expression in various groups of H9c2 cells after DLA treatment with or without SIRT1 siRNA (n = 6). **D**, Complex I activity in H9c2 cells from various groups based on the slope of increase in OD at 450 nm. **E**, Mitochondrial ROS estimated by DHR123 fluorescence in H9c2 cells of various groups. **F**, CCK-8 assay of H9c2 cells showing the cell viability in different groups. Results are presented as mean ± S.E.M (n = 6) * *p* < 0.05 *vs.* control group, # *p* < 0.05 *vs.* H/R group, & *p* < 0.05 *vs.* H/R+DLA group.

In order to confirm the involvement of SIRT1 in DLA alleviating I/R-induced cardiomyocyte injury, SIRT1 expression was knocked down by treatment with siRNA in H9c2 cells exposing to hypoxia/reoxygenation (H/R). As shown in Fig. 8A,B, SIRT1siRNA considerably reduced the protein level of SIRT1 under normal or H/R condition.

As an important subunit of Complex I, NDUFA10 in H9c2 cells was examined by western blotting assay, as shown in Fig. 8C. As expected, H/R led to a decrease in NDUFA10 while treatment with DLA restrained NDUFA10 expression after H/R. SIRT1 knocked down by siRNA decreased NDUFA10 after DLA treatment in H/R condition. Mitochondrial Complex I activity in H9c2 cells was then determined, and the results are shown in Fig. 8D in which OD at 450 nm increased over time being proportional to Complex I activity. Evidently, increased Complex I activity induced by DLA treatment after H/R was remarkably reversed by SIRT1siRNA.

Complex I activity is necessary for mitochondrial respiration, which maintains intracellular normal free radical level. Therefore, we evaluated the basal and inducible mitochondrial ROS level in H9c2 cells using molecular probe DHR 123. As shown in Fig. 8E, the ROS level was considerably increased after H/R compared to control cells. DLA treatment reduced the H/R-elevated ROS level, while SIRT1 knockdown reversed the antioxidative role of DLA, leading to a higher ROS in H9c2 cells.

The results of CCK-8 assay (Fig. 8F) showed that at the end of reoxygenation, the viability of H9c2 cells was decreased to approximate a half of control group. Treatment with DLA attenuated the H/R-induced depression in cell survival. DLA treatment of cells with knocked down SIRT1 showed a remarkably reduced cell viability compared with DLA group without SIRT1siRNA. Above results indicated that SIRT1 played an important role in DLA restoring NDUFA10 expression and Complex I activity after H/R, as well as in maintaining intracellular redox state and cell viability.

## Discussion

Our results demonstrated for the first time that DLA could restore NDUFA10 expression and Complex I activity after I/R, which is probably attributable to the ability of DLA to bind to SIRT1 and preserve SIRT1 activity, which eventually results in reduced infarct size, improved LV function and better recovery of MBF (Supplementary Fig. S5).

Our 2D-DIGE result provided novel insights into the role of mitochondria in DLA mediated anti-apoptotic effect in response to I/R, showing that NDUFA10, among others, was better maintained by DLA after I/R injury. More importantly, restoration of NDUFA10 by DLA was accompanied by an improved mitochondrial Complex I activity after I/R. Under the conditions of hypoxia/reoxygenation^30^, ischemic and I/R^31^, a decreased mitochondrial Complex I activity was observed. Given to the central role of Complex I in electron transport and oxidative phosphorylation, restoration of Complex I activity is expected to be one of the most promising strategies for reducing ROS and increasing ATP production after I/R. The results of present study, as shown by the decrease in MDA level and increase in ATP content by DLA, supported the above speculation, suggesting a beneficial role of DLA-provoked Complex I activity.

An emerging concept is that blockade of ETC, including Complex I, during ischemia is cardioprotective^7^. One possibility accounting for this discrepancy may be that mitochondrial damage caused by ETC mainly occurs in ischemia period, the continuation of enhanced mitochondrial aerobic respiration without oxygen results in deletion of cardiolipin, leading to loss of cytochrome *c* and the onset of mitochondrial permeability transition, which taken together promote myocardial cell apoptosis^32^,^33^. This is why most, if not all, the cardioprotective roles for blockage of Complex I are achieved by intervention immediately before ischemia or during ischemia. In the present study, DLA was administrated 1 min before reperfusion until the end of reperfusion. During this phase, sufficient oxygen is available that requires a dramatic increase in Complex I activity to drive the electron passing through ETC. Another possibility is that DLA imposed effect not directly on Complex I but on SIRT1, which modulated a range of proteins, including NDUFA10. The cardioprotective role of DLA observed in current study was a collective outcome of the activation of these proteins.

Study has demonstrated that SIRT1 mutant mice exhibit notable developmental defects in the heart^34^. Moderate increased SIRT1 was found to protect the heart against oxidative stress by upregulating antioxidant expressions^35^. Overexpression of SIRT1 was revealed to prevent myocardium apoptosis in response to serum starvation^36^. Furthermore, cardiac-specific overexpression of SIRT1 was also shown to reduce infarct size and improve left ventricular functions after I/R, whereas cardiac-specific SIRT1 knockout mice were more susceptible to I/R injury^29^. Thus, SIRT1 is considered to be an effective target in alleviating I/R induced injury.

SIRT1 has been reported to increase transcription of antioxidants^29^. Consistent with this finding, we observed that MnSOD content was enhanced in response to the administration of DLA. DLA is well known for its antioxidant activity, however, the underlying mechanisms are poorly understood. The results of present study implied that antioxidative effect of DLA may be attributable to maintenance of Complex I integrity and activity, which prevented electron leakage from ETC, as well as to upregulation of MnSOD that was mediated by DLA binding to and activating SIRT1.

Our results revealed DLA was able to bind to SIRT1 *in vitro* in a dose dependent manner, possibly depending on the presence of phenolic hydroxyl groups. However, we can not conclude that phenolic hydroxyl is the exact binding group of DLA to SIRT1. More experiments need to be carried out to investigate the roles of other groups in addition to phenolic hydroxyl. Besides, replacing phenolic hydroxyl of DLA into methoxyl or hydroxyl might lead to an alteration in conformation and physic-chemical property of DLA, which in turn may affect its binding affinity for SIRT1. Especially, structural evidence is required to confirm the biding of DLA to SIRT1, and the binding affinity of DLA to SIRT1 needs to be assessed *in vivo*. Thus, the exact mechanism responsible for the interaction between DLA and SIRT1 remains to be clarified.

We have identified DLA as a regulator of mitochondrial function probably through modulating SIRT1 activity during I/R injury in the heart. However it remains unclear how SIRT1 achieved the transcriptional regulation of NDUFA10 mRNA after DLA administration. Several lines of evidence suggest that PGC-1α serves as the downstream effector of SIRT1 in upregulating mitochondrial respiration genes^18^. Thus, PGC-1α might be responsible for upregulating NDUFA10 expression in the presence of DLA treatment. Nevertheless, more works are required to clarify the signaling pathway which links up SIRT1 to NDUFA10 for DLA to attenuate the Complex I dysfunction after I/R.

### Summary

In the present study, we confirmed the cardioprotective role of DLA against I/R injury and revealed the ability of DLA to bind to and activate SIRT1, resulting in enhanced expression of NDUFA10, Complex I activity and mitochondrial function after I/R. These results provide clue for identification of the signaling pathway thereby DLA exerts its beneficial effects, as well as for designing novel medicine to cope with I/R-induced cardiac injury.

## Methods

### Animals

Male Sprague-Dawley (SD) rats weighing 240-260 g were purchased from the Animal Center of Peking University Health Science Center (Certificate no. SCXK (Jing) 2006-0008). All experimental procedures involving animals were approved by Peking University Biomedical Ethics Committee Experimental Animal Ethics Branch (LA2010-001), complying with the Guide for the Care and Use of Laboratory Animals (NIH publication no. 85-23, 1996).

### I/R model establishing and drug administration

Rats were anesthetized by intraperitoneal injection of sodium pentobarbital (60 mg/kg) and maintained by 1% isoflurane inhalation. Anesthetic monitoring was achieved by a pinch on the toe. I/R animal model was established as previously described^19^. In SHAM group, the rats underwent the same procedure, except for tightening the ligature. A polyethylene catheter was inserted into left femoral vein for administration of DLA (Kun Ming Feng-Shan-Jian Medical Company, Yunnan, China), Compound A, Compound B or normal saline (NS). DLA, Compound A and Compound B were dissolved in 2 ml NS, and 0.5 ml was given intravenously 1 min before reperfusion, and the remaining was continuously administrated till the end of reperfusion at a speed of 1 ml/h. DLA was given at a dose of 1.25 mg/kg, 2.5 mg/kg, 5 mg/kg, 10 mg/kg, or 20 mg/kg. Compound A and Compound B were injected at the dose of 5 mg/kg.

### 2,3,5-triphenyltetrazolium chloride and Evans blue staining

At the end of reperfusion, the left descending artery (LAD) was re-ligated, and 2 ml of 4% Evans blue (Sigma Aldrich, Missouri, USA) was injected into the right femoral vein via intravenous catheter to delineate the area at risk (AAR). Then the heart was excised at a 5 mm level above the apex cordis and sliced transversely into five sections (1 mm thick). The slices were incubated in 2% 2,3,5-triphenyltetrazolium chloride (TTC) (AMRESCO, Ohio, USA) at 37 °C for 15 min to identify the infarction area (IA). AAR, IA and left ventricular area (LA) were analyzed by Image-Pro Plus 5.0 software (Media Cybernetic, Maryland, USA).

### Echocardiographic analysis

The left ventricular wall thickness and heart function were evaluated at the third week after myocardial I/R, using a Vevo 770 High-Resolution Imaging System (Vevo 770, Visual Sonics Inc, Toronto, Canada) with a 17.5 MHz linear array transducer (model 716). The parameters assessed were as follows: left ventricular internal diameter at end-diastole/end-systole (LVIDd/LVIDs), left ventricular anterior wall at end-diastole/end-systole (LVAWd/LVAWs), left ventricular volume at end-diastole/end-systole (LV Vold/LV Vols), left ventricular mass (LV Mass), left ventricular posterior wall at end-diastole/end-systole (LVPWd/LVPWs), left ventricle ejection fraction (EF%), left ventricle fractional shortening (FS%) and left ventricular fractional area change (FAC%).

### SIRT1 inhibitor administration

SIRT1 inhibitors sirtinol and EX-527 were injected intravenously 45 min before DLA administration. The dose of sirtinol used in the experiments was 1.2 mg/kg, 2.4 mg/kg, or 4.8 mg/kg, and the dose of EX-527 was 1.2 mg/kg, 4 mg/kg, or 12 mg/kg. SIRT1 inhibitors were first dissolved in stock solutions of DMSO, and for treatment, the final concentration of DMSO was 0.2% (v/v). In groups without inhibitor administration, DMSO at the same final concentration was given to rats. No obvious adverse effects were observed in DMSO treated groups.

### Detection of deacetylase activity

After ischemic heart tissue was collected and homogenized, proteins were extracted. Tissue Sirtuin activity was detected using a SIRT1 fluorometric assay kit (Enzo, New York, USA), according to manufacturer instruction. Samples with 100 g of total protein were incubated with Fluor de Lys-SIRT1 substrate (100 μM), NAD^+^ (50 μM) at 37 °C for 60 min, and 50 μl mixture of developer and nicotinamide (2 mM) was added by pipetting. Fluorescence kinetics was monitored for 1 h with measuring at 360 nm (excitation) and 460 nm (emission) at 37 °C every 5 minutes^37^.

### Electron microscopy

After reperfusion, hearts were fixed by infusion with a fixative containing 5% paraformaldehyde and 2% glutaraldehyde for 40 min. Cardiac tissue was excised as described before, and trimmed into 1 mm^3^ and fixed overnight in 3% glutaraldehyde at 4 °C. Trimmed tissues were post-fixed by 1% osmium tetraoxide for 2 h and prepared for thin sectioning. The thin sections were observed with a transmission electron microscope (JEM 1230, JEOL, Tokyo, Japan).

### 2-dimension fluorescence difference gel electrophoresis

The samples were harvested as described before and prepared with a 2-dimension (2D)-Clean up Kit (GE Healthcare, Buckinghamshire, UK). Equal amounts of proteins from all samples were pooled together as the internal standard labeled with Cy2. Fifty micrograms of lysates were labeled with 400 pmol of Cy3 or Cy5. The DIGE experimental design was as follows: Gel 01, internal standard (Cy2), SHAM+NS group (Cy3), I/R+NS group (Cy5); Gel 02, internal standard (Cy2), I/R+DLA group (Cy3), SHAM+DLA group (Cy5). Immobilized pH gradient (IPG) strips (24 cm, pH 3-10, and nonlinear) were prepared with labeled samples as previously reported^38^. Then IPG strips were placed on the top of 12% polyacrylamide gels, and electrophoresed for ∼10 hours at 30 mA per gel using an Ettan DALT Six system (GE Healthcare, Buckinghamshire, UK). Then, protein spots were selected and identified as described previously^39^.

### Surface plasmon resonance

Carboxymethylated 5 (CM5) sensor chip was docked into the BIAcore 3000 (GE Healthcare, Buckinghamshire, UK), and prepared as previously reported^40^. Human recombinant SIRT1 (Sigma, Missouri, USA) was immobilized on CM5 sensor chip by injecting 40 μl of SIRT1 (40 μg/ml in 10 mM sodium acetate, pH 4.0) at the rate of 5 μl/min. DLA, Compound A, and Compound B were prepared as a 20 mM solution in running buffer before the experiment, and two fold diluted by running buffer into 10 mM, 5 mM, 2.5 mM, 1.25 mM, 625 μM, 312.5 μM before injection. Analytes were injected at 30 μl/min over SIRT1 and control sensor chip. Equilibrium dissociation (K_D_) was calculated by fitting a 1:1 Langmuir model using the BIA evaluation 4.1 software (GE Healthcare, Buckinghamshire, UK)

### Biolayer interferometry

All Biolayer interferometry (BLI) experiments were performed on an Octet Red instrument (ForteBio Inc., California, USA) at 25 °C. Super Streptavidin (SSA) biosensors were loaded with biotinylated SIRT1 (20 μg/ml) antibody followed by recombinant human SIRT1 (50 μg/ml, 200 μl). Association of DLA (20 mM, 10 mM, 5 mM, 2.5 mM) and SIRT1 was observed by immersing biosensors in wells containing DLA solution for 120 s. Disassociation was monitored after transfer of the biosensors into PBS for 240 s. K_D_ was analyzed by 1:1 binding global fit model using the Octet data analysis software 6.3 (ForteBio Inc., California, USA)^41^.

### Cell culture and H/R model

H9c2, a rat cardiac myoblast cell line (ATCC, Maryland, USA), was cultured in Dulbecco’s Modified Eagle’s Medium (Invitrogen, Grand Island, UK) containing 4 mM L-glutamine, 4.5 g/L glucose, and 10% fetal bovine serum (Invitrogen, Grand Island, UK) at 37 °C in a humidified incubator with 95% air and 5% CO_2_. The cells were subjected to hypoxia/reoxygenation (H/R) by incubation in a microaerophilic system (Thermo Scientific, Waltham, MA) at 5% CO_2_ and 1% O_2_ for 12 h followed by normoxic conditions for 6 h with or without 0.1 mM DLA treatment.

### Small interfering RNA transfection

When 50%-60% confluence was reached, H9c2 cells were transfected with 20 μM SIRT1 small interfering RNA (siRNA) or nontargeting control siRNA complexed with Lipofectamine 2000 (Invitrogen, Carlsbad, CA), according to manufacturer’s instructions. The sense sequences of SIRT1 siRNA was as follows: 5’- CAGGAAUCCAAAGGAUAAUTT-3’ (Genepharma, Shanghai, China).

### Statistical Analysis

All data were expressed as mean ± S.E.M. Statistical analysis was performed using one-way ANOVA followed by Newman-Keuls test or using two-way ANOVA (myocardial blood flow (MBF), left ventricular (LV) developed pressure (max), LV developed pressure (min) and LV systolic pressure) followed by Bonferroni for multiple comparisons. A *p* value less than 0.05 was considered to be statistically significant.

## Additional Information

**How to cite this article**: Yang, X.-Y. *et al.* 3, 4-dihydroxyl-phenyl lactic acid restores NADH dehydrogenase 1 α subunit 10 to ameliorate cardiac reperfusion injury. *Sci. Rep.* **5**, 10739; doi: 10.1038/srep10739 (2015).

## Supplementary Material

Supplementary Information

## References

[b1] Yellon DM, H. D. Myocardial reperfusion injury. N Engl. J. Med. 357, 1121–1135 (2007). 17855673 10.1056/NEJMra071667

[b2] Boden W. E. *et al.* Optimal medical therapy with or without PCI for stable coronary disease. N Engl J Med 356, 1503–1516, 10.1056/NEJMoa070829 (2007). 17387127

[b3] Fan Q. *et al.* Inhibition of Fas-associated death domain-containing protein (FADD) protects against myocardial ischemia/reperfusion injury in a heart failure mouse model. PLoS One 8, e73537, 10.1371/journal.pone.0073537 (2013). 24058479 PMC3772851

[b4] Katz M. Y. *et al.* Three-dimensional myocardial scarring along myofibers after coronary ischemia-reperfusion revealed by computerized images of histological assays. Physiol Rep 2, 10.14814/phy2.12072 (2014). PMC4187547 25347856

[b5] Borutaite V., Jekabsone A., Morkuniene R. & Brown G. C. Inhibition of mitochondrial permeability transition prevents mitochondrial dysfunction, cytochrome c release and apoptosis induced by heart ischemia. J Mol Cell Cardiol 35, 357–366 (2003). 12689815 10.1016/s0022-2828(03)00005-1

[b6] Balaban R. S., Nemoto S. & Finkel T. Mitochondria, oxidants, and aging. Cell 120, 483–495, 10.1016/j.cell.2005.02.001 (2005). 15734681

[b7] Chen Q., Camara A. K., Stowe D. F., Hoppel C. L. & Lesnefsky E. J. Modulation of electron transport protects cardiac mitochondria and decreases myocardial injury during ischemia and reperfusion. Am J Physiol Cell Physiol 292, C137–147, 10.1152/ajpcell.00270.2006 (2007). 16971498

[b8] Turrens J. F. & Boveris A. Generation of superoxide anion by the NADH dehydrogenase of bovine heart mitochondria. Biochem J 191, 421–427 (1980). 6263247 10.1042/bj1910421 PMC1162232

[b9] Meng C. *et al.* Alterations of mitochondrial enzymes contribute to cardiac hypertrophy before hypertension development in spontaneously hypertensive rats. J Proteome Res 8, 2463–2475, 10.1021/pr801059u (2009). 19265432

[b10] Olsson A. H. *et al.* Decreased expression of genes involved in oxidative phosphorylation in human pancreatic islets from patients with type 2 diabetes. Eur J Endocrinol 165, 589–595, 10.1530/EJE-11-0282 (2011). 21775499 PMC3178933

[b11] Hoefs S. J. *et al.* NDUFA10 mutations cause complex I deficiency in a patient with Leigh disease. Eur J Hum Genet 19, 270–274, 10.1038/ejhg.2010.204 (2011). 21150889 PMC3061993

[b12] Yamaguchi M., Belogrudov G. I., Matsuno-Yagi A. & Hatefi Y. The multiple nicotinamide nucleotide-binding subunits of bovine heart mitochondrial NADH:ubiquinone oxidoreductase (complex I). Eur J Biochem 267, 329–336 (2000). 10632702 10.1046/j.1432-1327.2000.00999.x

[b13] Gurd B. J. *et al.* Nuclear SIRT1 activity, but not protein content, regulates mitochondrial biogenesis in rat and human skeletal muscle. Am J Physiol Regul Integr Comp Physiol 301, R67–75, 10.1152/ajpregu.00417.2010 (2011). 21543634

[b14] Brunet A. *et al.* Stress-dependent regulation of FOXO transcription factors by the SIRT1 deacetylase. Science 303, 2011–2015, 10.1126/science.1094637 (2004). 14976264

[b15] Haigis M. C. & Guarente L. P. Mammalian sirtuins--emerging roles in physiology, aging, and calorie restriction. Genes Dev 20, 2913–2921, 10.1101/gad.1467506 (2006). 17079682

[b16] Haigis M. C. & Sinclair D. A. Mammalian sirtuins: biological insights and disease relevance. Annu Rev Pathol 5, 253–295, 10.1146/annurev.pathol.4.110807.092250 (2010). 20078221 PMC2866163

[b17] Gerhart-Hines Z. *et al.* Metabolic control of muscle mitochondrial function and fatty acid oxidation through SIRT1/PGC-1alpha. EMBO J 26, 1913–1923, 10.1038/sj.emboj.7601633 (2007). 17347648 PMC1847661

[b18] Lagouge M. *et al.* Resveratrol improves mitochondrial function and protects against metabolic disease by activating SIRT1 and PGC-1alpha. Cell 127, 1109–1122, 10.1016/j.cell.2006.11.013 (2006). 17112576

[b19] Zhao N. *et al.* Cardiotonic pills, a compound Chinese medicine, protects ischemia-reperfusion-induced microcirculatory disturbance and myocardial damage in rats. Am J Physiol Heart Circ Physiol 298, H1166–1176, 10.1152/ajpheart.01186.2009 (2010). 20118406

[b20] Wei X. H. *et al.* Treatment of Cardiotonic Pills((R)) after Ischemia-Reperfusion Ameliorates Myocardial Fibrosis in Rats. Microcirculation , 10.1111/micc.12002 (2012). 22913380

[b21] Lam F. F., Yeung J. H., Chan K. M. & Or P. M. Relaxant effects of danshen aqueous extract and its constituent danshensu on rat coronary artery are mediated by inhibition of calcium channels. Vascul Pharmacol 46, 271–277, 10.1016/j.vph.2006.10.011 (2007). 17188580

[b22] Wu L. *et al.* Proliferative inhibition of danxiongfang and its active ingredients on rat vascular smooth muscle cell and protective effect on the VSMC damage induced by hydrogen peroxide. J Ethnopharmacol 126, 197–206, 10.1016/j.jep.2009.08.045 (2009). 19735709

[b23] Zhao G. R. *et al.* Characterization of the radical scavenging and antioxidant activities of danshensu and salvianolic acid B. Food Chem Toxicol 46, 73–81, 10.1016/j.fct.2007.06.034 (2008). 17719161

[b24] Ding M. & Yuan Y. J. Study on the mechanisms of an extract of Salvia miltiorrhiza on the regulation of permeability of endothelial cells exposed to tumour necrosis factor-alpha. J Pharm Pharmacol 59, 1027–1033, 10.1211/jpp.59.7.0016 (2007). 17637199

[b25] Wu L., Qiao H., Li Y. & Li L. Protective roles of puerarin and Danshensu on acute ischemic myocardial injury in rats. Phytomedicine 14, 652–658, 10.1016/j.phymed.2007.07.060 (2007). 17870452

[b26] Zhao B. L., Jiang W., Zhao Y., Hou J. W. & Xin W. J. Scavenging effects of salvia miltiorrhiza on free radicals and its protection for myocardial mitochondrial membranes from ischemia-reperfusion injury. Biochem Mol Biol Int 38, 1171–1182 (1996). 8739039

[b27] Schoenenberger C. A., Mannherz H. G. & Jockusch B. M. Actin: from structural plasticity to functional diversity. Eur J Cell Biol 90, 797–804, 10.1016/j.ejcb.2011.05.002 (2011). 21820202

[b28] Koopman W. J. *et al.* Mammalian mitochondrial complex I: biogenesis, regulation, and reactive oxygen species generation. Antioxid Redox Signal 12, 1431–1470, 10.1089/ars.2009.2743 (2010). 19803744

[b29] Hsu C. P. *et al.* Silent information regulator 1 protects the heart from ischemia/reperfusion. Circulation 122, 2170–2182, 10.1161/CIRCULATIONAHA.110.958033 (2010). 21060073 PMC3003297

[b30] Hardy L., Clark J. B., Darley-Usmar V. M., Smith D. R. & Stone D. Reoxygenation-dependent decrease in mitochondrial NADH:CoQ reductase (Complex I) activity in the hypoxic/reoxygenated rat heart. Biochem J 274 (Pt 1), 133–137 (1991). 1900416 10.1042/bj2740133 PMC1149930

[b31] Paradies G. *et al.* Decrease in mitochondrial complex I activity in ischemic/reperfused rat heart: involvement of reactive oxygen species and cardiolipin. Circ Res 94, 53–59, 10.1161/01.RES.0000109416.56608.64 (2004). 14656928

[b32] Lesnefsky E. J. *et al.* Blockade of electron transport during ischemia protects cardiac mitochondria. J Biol Chem 279, 47961–47967, 10.1074/jbc.M409720200 (2004). 15347666

[b33] Yoshioka J. *et al.* Deletion of thioredoxin-interacting protein in mice impairs mitochondrial function but protects the myocardium from ischemia-reperfusion injury. J Clin Invest 122, 267–279, 10.1172/JCI44927 (2012). 22201682 PMC3248280

[b34] Cheng H. L. *et al.* Developmental defects and p53 hyperacetylation in Sir2 homolog (SIRT1)-deficient mice. Proc Natl Acad Sci USA 100, 10794–10799, 10.1073/pnas.1934713100 (2003). 12960381 PMC196882

[b35] Alcendor R. R. *et al.* Sirt1 regulates aging and resistance to oxidative stress in the heart. Circ Res 100, 1512–1521, 10.1161/01.RES.0000267723.65696.4a (2007). 17446436

[b36] Alcendor R. R., Kirshenbaum L. A., Imai S., Vatner S. F. & Sadoshima J. Silent information regulator 2alpha, a longevity factor and class III histone deacetylase, is an essential endogenous apoptosis inhibitor in cardiac myocytes. Circ Res 95, 971–980, 10.1161/01.RES.0000147557.75257.ff (2004). 15486319

[b37] Chabi B., Adhihetty P. J., O’Leary M. F., Menzies K. J. & Hood D. A. Relationship between Sirt1 expression and mitochondrial proteins during conditions of chronic muscle use and disuse. J Appl Physiol 107, 1730–1735, 10.1152/japplphysiol.91451.2008 (2009). 19797682

[b38] Jimenez C. R. *et al.* Proteomics of the injured rat sciatic nerve reveals protein expression dynamics during regeneration. Mol Cell Proteomics 4, 120–132, 10.1074/mcp.M400076-MCP200 (2005). 15509515

[b39] Zhang X. *et al.* Proteomic analysis of individual variation in normal livers of human beings using difference gel electrophoresis. Proteomics 6, 5260–5268, 10.1002/pmic.200600006 (2006). 16947120

[b40] Zhang J. *et al.* Aquaporin-1 translocation and degradation mediates the water transportation mechanism of acetazolamide. PLoS One 7, e45976, 10.1371/journal.pone.0045976 (2012). 23029347 PMC3448731

[b41] Cattepoel S., Hanenberg M., Kulic L. & Nitsch R. M. Chronic intranasal treatment with an anti-Abeta(30-42) scFv antibody ameliorates amyloid pathology in a transgenic mouse model of Alzheimer’s disease. PLoS One 6, e18296, 10.1371/journal.pone.0018296 (2011). 21483675 PMC3071717

