# Supplementary material for: 3, 4-dihydroxyl-phenyl lactic acid restores NADH dehydrogenase 1 α subunit 10 to ameliorate cardiac reperfusion injury
Source: Sci Rep. 2015 Jun 1;5:10739. doi: 10.1038/srep10739 (PMC5377067; doi:10.1038/srep10739)
Supplement: Supplementary Information [file srep10739-s1.doc]

**3, 4-dihydroxyl-phenyl lactic acid restores NADH dehydrogenase 1 α subunit 10 to ameliorate cardiac reperfusion injury**

Xiao-Yuan Yang; Ke He; Chun-Shui Pan; Quan Li; Yu-Ying Liu; Li Yan; Xiao-Hong Wei; Bai-He Hu; Xin Chang; Xiao-Wei Mao; Dan-Dan Huang; Li-Jun Wang; Shui-Wang Hu; Yong Jiang; Guo-Cheng Wang; Jing-Yu Fan; Tai-Ping Fan; Jing-Yan Han

**Supplementary Materials:**

Materials and Methods

Tables S1-S3

Figures S1-S5

**Materials and Methods:**

1. Agents

DLA was purchased from Kun Ming Feng-Shan-Jian Medical Company (Yunnan, China). TTC obtained from AMRESCO Co., Ltd. (Solon, OH, USA) was dissolved in phosphate buffer at a concentration of 2.0%. Evans Blue was from Sigma Chemical Co., Ltd. (St Louis, Missouri, USA).

2. Myocardial Blood Flow

After left thoracotomy, MBF was determined using Laser-Doppler Perfusion Imager (PeriScan PIM3, Perimed, Stockholm, Sweden) equipped with a computer. MBF was recorded before ischemia (baseline), 30 min after ischemia, and 10, 30, 60, 90 min of reperfusion. All images were analyzed by LDPIwin 3.1 software (Perimed, Stockholm, Sweden). Results were expressed as percentages of the baseline .

3. Hemodynamic examination

A polyethylene catheter connected with bio-function experiment system (BL-420F, Chengdu Taimen technology Co., Ltd., Chengdu, China) was inserted into the left ventricle through right common carotid artery. Upon stabilization, cardiac ischemia was induced. Hemodynamic parameters, including LV developed pressure (max), LV developed pressure (min), LV end diastolic pressure and mean artery pressure, etc. were continuously recorded till the end of reperfusion. After 90 min of reperfusion, the catheter was removed from left ventricle to right common carotid artery to measure arterial pressure.

4. Histological evaluation of cardiac tissues

Heart was excised at 90 min of the reperfusion, fixed in 4% paraformaldehyde for 48 h. Then, 5 μm parafﬁn-embedded sections were prepared and stained with hematoxylin and eosin (HE). The images were captured by a conventional microscope (Digital Sight DS-5M-U1, Nikon, Tokyo, Japan) connected with a digital camera (SZ-40, Olympus, Tokyo, Japan).

5. F-actin staining

The paraffin sections were deparaffinized, rehydrated and treated with 0.01 M sodium citrate for antigen retrieval. Sections were washed by PBS for three times and incubated with rhodamine phalloidine (1:40, R415, Invitrogen, California, USA) in a humidified dark box for 1 hour at 37 ℃, then washed with PBS for 3 times. In order to label nucleus, sections were incubated with Hoechst 33342 (1:100, Molecular Probes) at room temperature for 10 min. Images were captured with a laser scanning confocal microscope (TCS SP5, Leica, Mannheim, Germany)

6. Myeloperoxidase (MPO) Activity

To assess neutrophil infiltration, MPO activity was detected in heart tissues. Paraffin tissue sections were stained with MPO antibody (Santa Cruz Biotechnology, California, USA) following the manufacturer’s instruction. Each slide was examined by a conventional microscope (Digital Sight DS-5M-U1, Nikon, Tokyo, Japan) connected with a digital camera (SZ-40, Olympus, Tokyo, Japan), and five visual ﬁelds were selected from each section for analysis of the protein expression using Image-Pro Plus software.

7. Detection of myocardium apoptosis

After 90 min of reperfusion, heart was harvested, fixed in 4% formalin for 48 hours and prepared for paraffin sectioning. The paraffin sections (5 μm) were rehydrated and treated with 0.01 M sodium citrate. TUNEL was performed to detect apoptotic cardiomyocytes with a cell death detection kit (Roche, Basel, Switzerland), according to the manufacturer’s protocol. Five fields were selected from the surrounding infarction areas of the left ventricle, total numbers of nuclei (blue) and the TUNEL positive nuclei (green) in each field were scored by a laser confocal microscope (Axiovert 200M, Carl Zeiss, Jena, Germany). Images were analyzed with Image-Pro Plus 5.0 software.

8. Western blotting

Ischemic heart tissue (150 mg) was collected from left ventricle after 90 min of reperfusion, and homogenized. Proteins were separated by SDS-polyacrylamide gel electrophoresis and transferred to polyvinylidene difluoride (PVDF) membrane. Non-specific binding sites were blocked by pre-incubating PVDF membrane with 5% skimmed milk in Tris-buffered saline Tween (TBS-T). Then, PVDF membranes were incubated overnight at 4 ℃ with the primary antibodies against GAPDH (1:3000, Cell Signaling Technology, Vermont, USA), Bcl-2 (1:1000, Cell Signaling Technology, Vermont, USA), Bax (1:1000, Cell Signaling Technology, Vermont, USA), cleaved-Caspase-3 (1:500, Cell Signaling Technology, Vermont, USA), NDUFA10 (1:200, Santa Cruz Biotechnology, California, USA), SIRT1 (1:8000, Invitrogen, California, USA), Ac-Foxo-1 (1:200, Santa Cruz Biotechnology, California, USA), MnSOD (1:300, Santa Cruz Biotechnology, California, USA). After rinsing with TBS-T for 3 times, PVDF membranes were incubated with secondary antibody (1:5000, Cell Signaling Technology, Vermont, USA) for 1 h at room temperature and washed by TBS-T for 3 times. Antibody binding was detected by enhanced chemiluminescence detection kit (APPLYGEN, Beijing, China). Bands were scanned and calculated by Bio-Rad Quantity One software for quantification (Bio-Rad, California, USA).

9. mRNA isolation and reverse transcription real-time polymerase chain reaction.

Ischemic cardiac tissues (40 mg) were harvested after 90 min of reperfusion, snap frozen in liquid nitrogen, and stored at -80 ℃. RNA was extracted using RNeasy Fibrous Tissue Mini Kit (QIAGEN, Hilden, Germany), according to the manufacturer protocol. RNA concentration and purity were assessed by Eppendorf Biophotometer Plus (Hamburg, Germany). RNA was applied for reverse transcription using a Revert Aid First Strand cDNA Synthesis Kit (Fermentas, Lifesciences, UK) to generate the first strand cDNA mix. The reaction included 5 μg total RNA, the oligo (dT) 18, nuclease-free water, 5×reaction buffer, ribolock RNase inhibitor, dNTP and ReverAidTM M-MuLV reverse transcriptase. Then the reverse transcriptase was inactivated by heating to 70 ℃ for 5 min and the mixture were cooled to 4 ℃ before polymerase chain reaction (PCR).

Real-time PCR analysis was performed to detect NDUFA10 mRNA levels, using a Maxima SYBR Green/Rox qPCR Master Mix (Fermentas, Lifesciences, UK) on a DNA Engine OPTICON Continuous Fluorescence Detector (Genetic Technologies, Inc., Miami, FL, USA). The primers for NDUFA10 were: forward, 5’- ACAGTCCTGGCTTTATGC-3’ and reverse 5’-CACTGCTTTCGGATGAAG-3’; the primers for GAPDH were: forward, 5’-GGCACAGTCAAGGCTGAGAATG-3’ and reverse 5’-ATGGTGGTGAAGACGCCAGTA-3’. The PCR reaction mixture (25 μl) included 2×Maxima SYBR Green/ROX qPCR Master Mix, reverse transcription product cDNA, forward and reverse primers, nuclease-free water. The reactions were incubated in a 96-well plate at 50 ℃ for 2 min, 95 ℃ for 10 min, followed by 40 cycles of 95 ˚C for 15 sec, 58 ˚C for 1 min and plate was read. All tests were performed in triplicate. The products were analyzed using 1% agarosegels with DNA marker.

10. Detection of Complex Ⅰ activity, MDA level and ATP, AMP, ADP content of cardiac tissue

Hearts were harvested after 90 min of reperfusion, ischemia regions of hearts were excised from left ventricle, snap frozen in liquid nitrogen and stored at -80 ℃ till use. Proteins were extracted by using a protein extraction kit (Applygen, Beijing, China), according to manufacturer’s instruction. Total protein concentration was detected with BCA protein assay kit (Applygen, Beijing, China) according to instruction. All tests were performed twice. Complex Ⅰ activity was analyzed via Complex Ⅰ Enzyme Activity Microplate Assay Kit (abcam, Cambridge, UK). The level of MDA was measured as an indicator of lipid peroxidation using Rat MDA Elisa Kit (GBD Ltd., Californis, USA). ATP, ADP and AMP content were detected by using Rat ATP ELISA Kit, Rat ADP ELISA Kit, and Rat AMP ELISA Kit (R&D, Minnesota, USA). All plates were analyzed on MULTISKAN MK3 [enzyme micro-plate reader](javascript:showjdsw('jd_t','j_')) (Thermo Fisher Scientific Inc., Illinois, USA), according to manufacturer’s instruction.

11. Cell viability assay

H9c2 cell viability was determined colorimetrically using Cell Counting Kit-8 (CCK-8) assay according to manufacturer’s instruction (DOJINDO, Kumamoto, Japan).

12. Measurement of intracellular ROS

Dihydrorhodamine 123 (DHR 123) (Sigma Aldrich, Missouri, USA) was used for determination of mitochondrial ROS levels. Briefly, H9c2 cells plated in 96 well plates were treated with oxidation sensitive DHR 123 at a concentration of 10 μM at 37°C for 60 min and the fluorescence of DHR 123 in cells was measured (λex—485 nm, λem—528 nm) using MULTISKAN MK3 [enzyme micro-plate reader](javascript:showjdsw('jd_t','j_')) (Thermo Fisher Scientific Inc., Illinois, USA). The values were expressed as optical density (OD) per 1×10^4^ cells.

**Tables S1-S3**

**
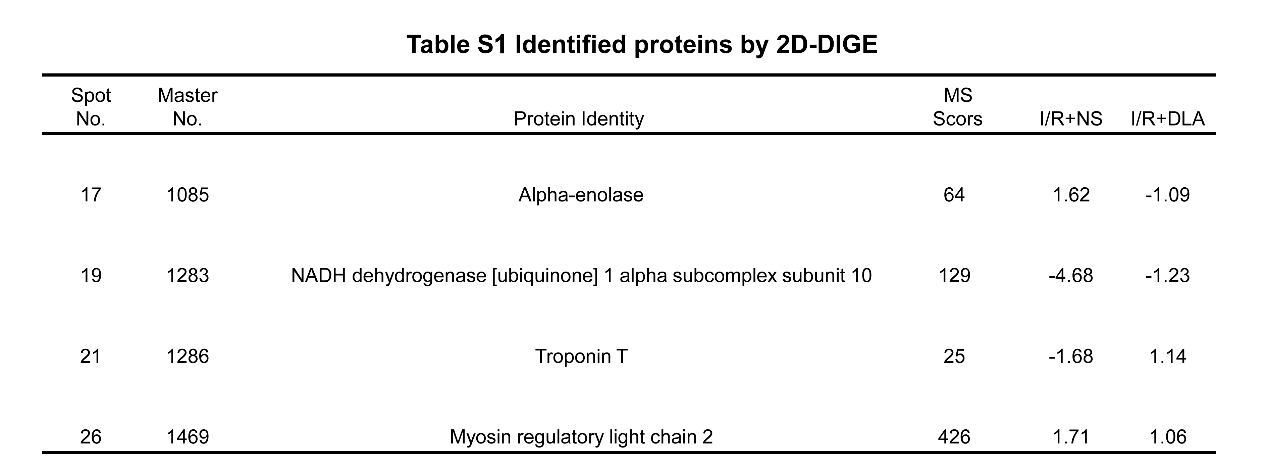
**

DLA administration represses I/R caused 4 proteins expression changes identified by 2D-DIGE. Summarized in the Table are the number of matched peptides, the protein identity, scores of protein identification and expression changes compared to sham group.


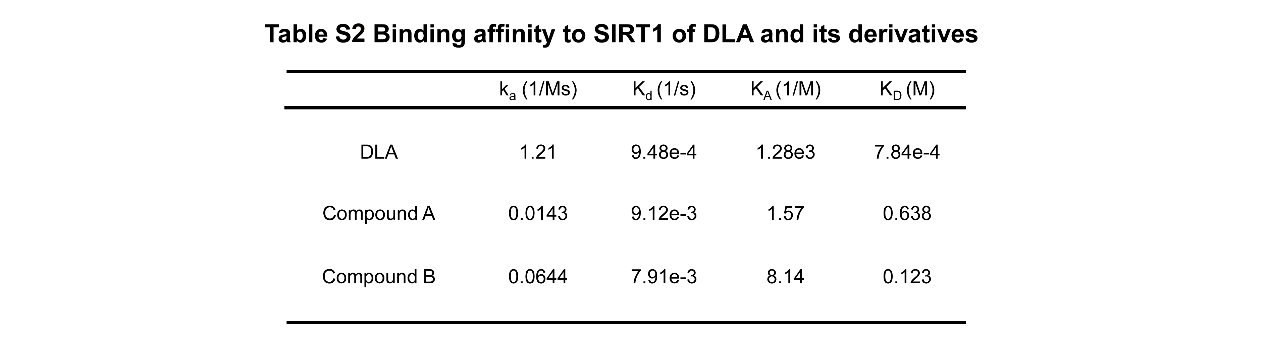


DLA derivatives, which were synthesized by replacing both phenolic hydroxyl of DLA into methoxyl (Compound A) or hydroxyl (Compound B), show lower affinity for SIRT1.
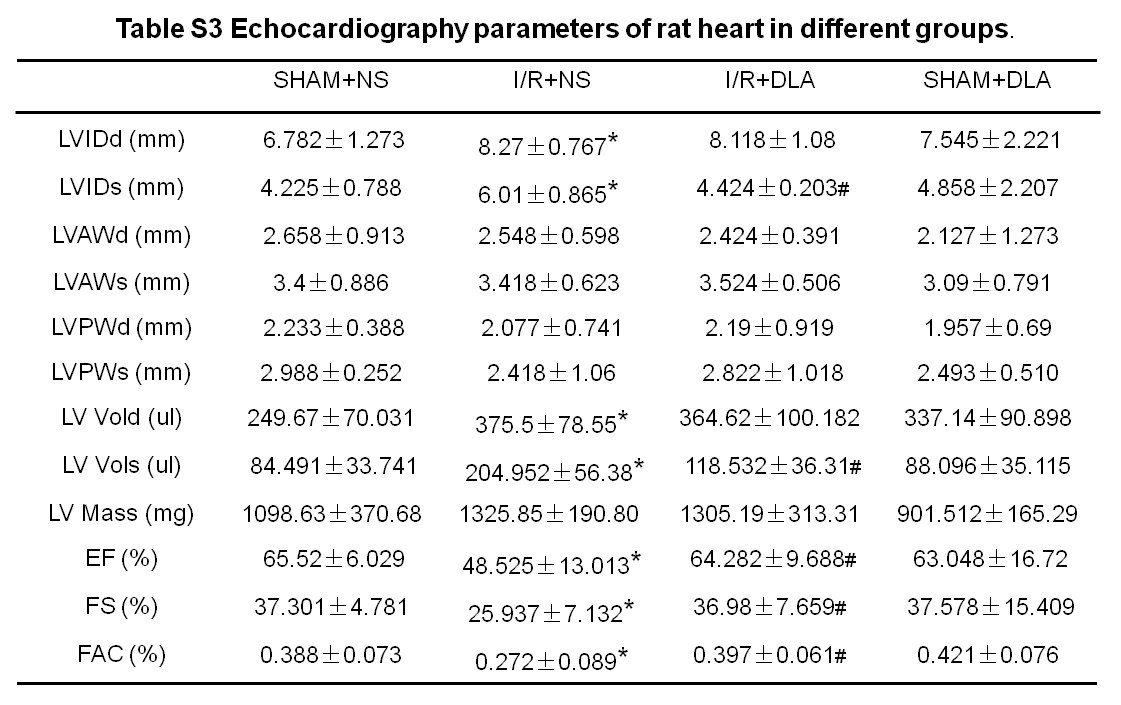


DLA administration represses I/R induced LVIDs, LV Vols, EF (%), FS (%) and FAC (%) changes identified by echocardiography. Results are presented as mean ± S.E.M (n = 6) * p < 0.05 vs. SHAM+NS group, # p < 0.05 vs. I/R+NS group.

**Figures S1-S5**

Figure S1

**
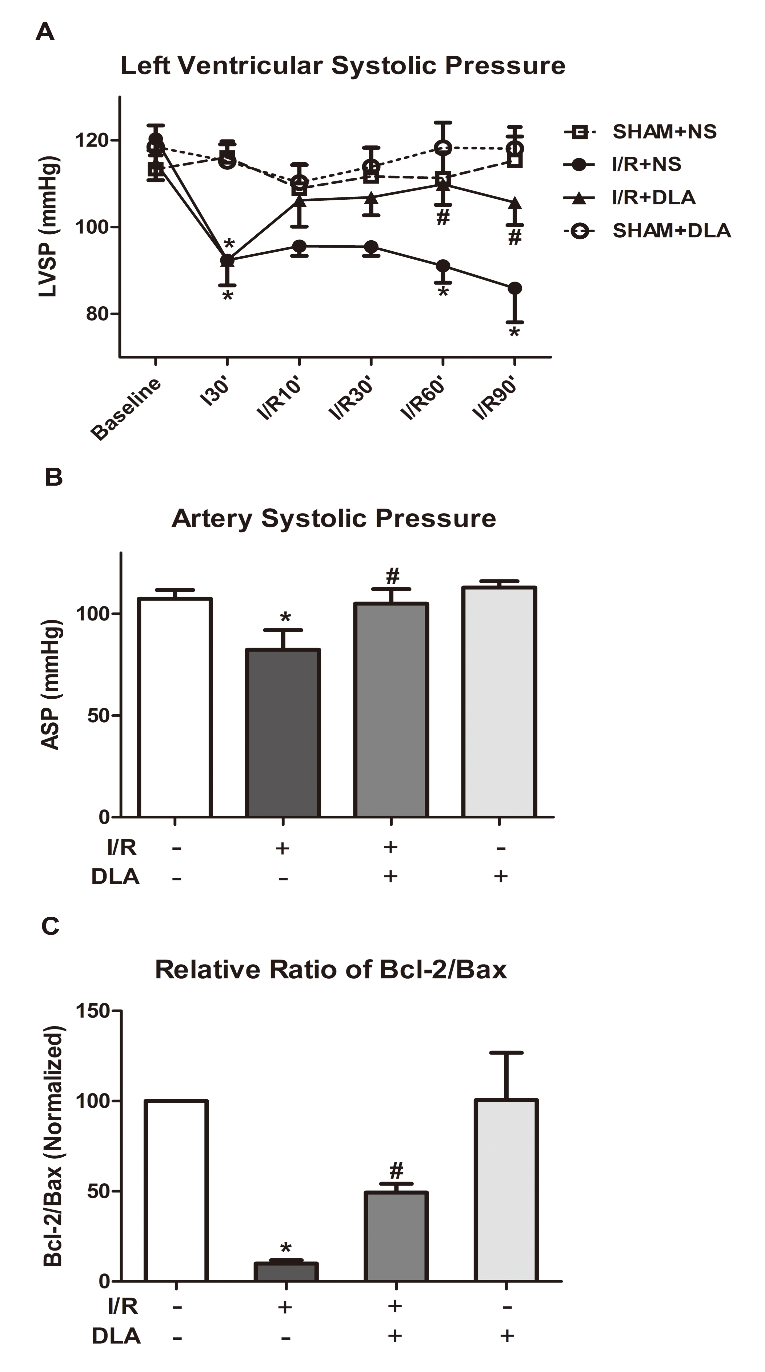
**

**Figure S1.** DLA improves heart function and increases Bcl-2/Bax in heart tissue after I/R. A, The results of LV systolic pressure at baseline, I 30’, I/R 10’, I/R 30’, I/R 60’ and I/R 90’ in four groups. B. Results of artery systolic pressure at 90 min of reperfusion in different groups. C, The ratio of the protein expression of Bcl-2 and Bax in various groups from 3 independent western blotting experiments. Results are presented as mean ± S.E.M (n = 6) * *p* < 0.05 *vs.* SHAM+NS group, # *p* < 0.05 *vs.* I/R+NS group.

Figure S2


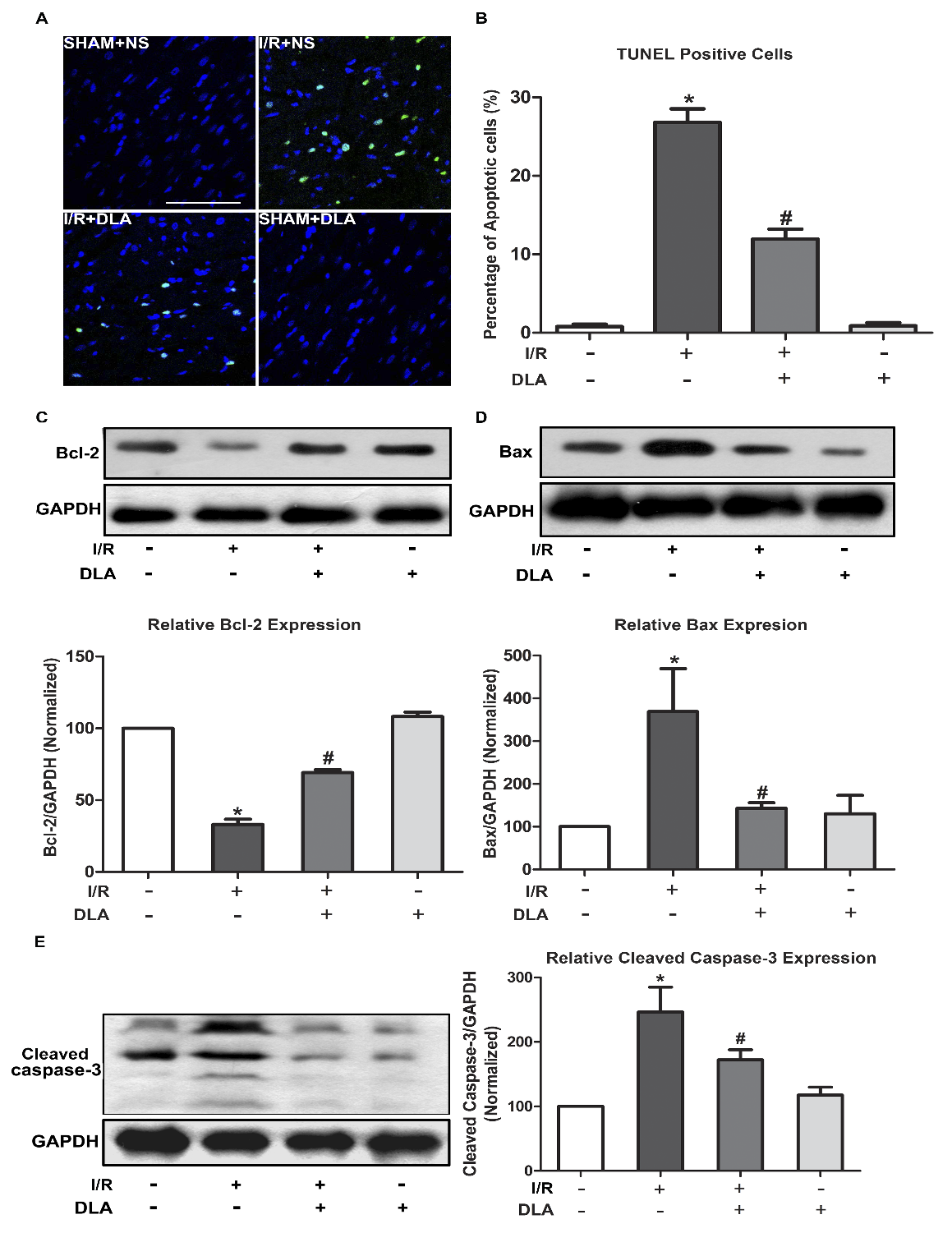


**Figure S2**. DLA administration reduces I/R-induced apoptosis. A, Representative photographs of myocardial sections from 4 groups stained with TUNEL and Hoechst. Apoptotic nuclei are identified by green and total nuclei by blue. Bar = 50 μm. B, Quantitative analysis of apoptotic nuclei in myocardium (n = 6). TUNEL-positive nuclei are expressed as a percentage of the total number of nuclei per field. C, D and E, Representative western blot bands and quantification of Bcl-2, Bax and Cleaved Caspase-3 in the myocardium of various groups, respectively. The gels have been run under the same experimental conditions. All the quantitative analyses of band intensities were undertaken based on the results from 3 independent experiments and normalized to GAPDH. Results are presented as mean ± S.E.M (n = 6),* *p* < 0.05 *vs.* SHAM+NS group, # *p* < 0.05 *vs.* I/R+NS group.

Figure S3


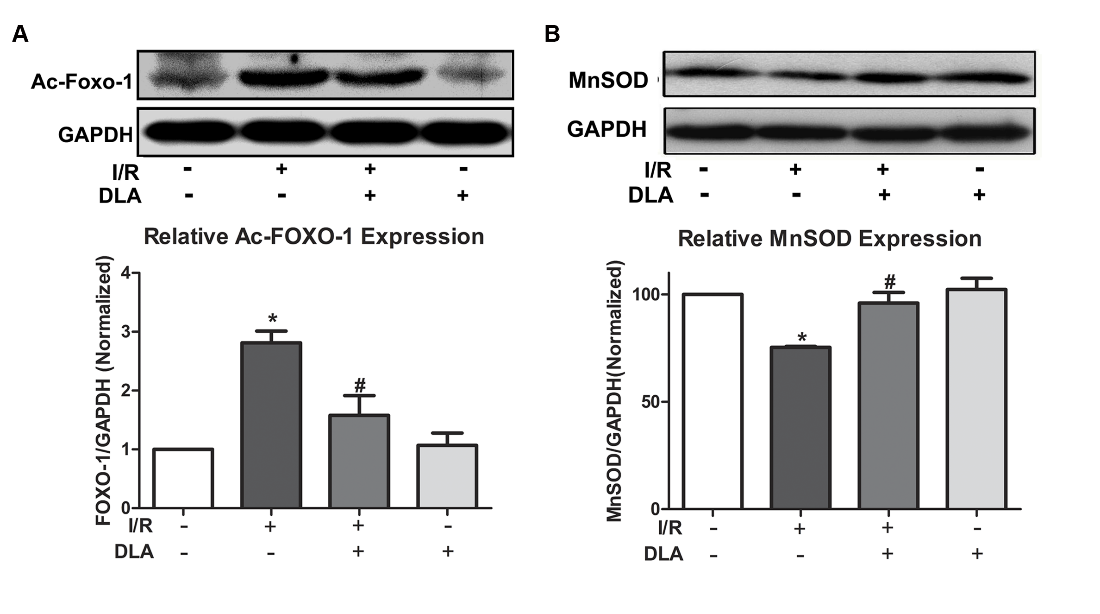


**Figure S3.** DLA administration decreases the acetylation level of Foxo-1A and increases the expression of MnSOD in heart tissue. A, Representative western blotting bands of Ac-Foxo-1 in various groups. The gels have been run under the same experimental conditions. The quantitative analyses of Ac-Foxo-1 band intensities were based on the data from 3 independent experiments and normalized to GAPDH. B, Representative western blotting bands of MnSOD in various groups. The gels have been run under the same experimental conditions. The quantification results for MnSOD band intensities were calculated based on the data from 3 independent experiments and normalized to GAPDH. Results are presented as mean ± S.E.M (n = 6) * *p* < 0.05 *vs.* SHAM+NS group, # *p* < 0.05 *vs.* I/R+NS group.

Figure S4


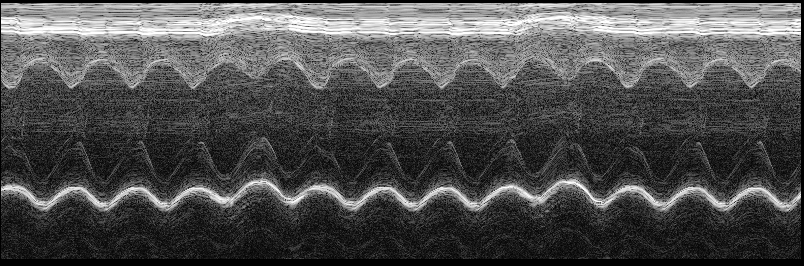

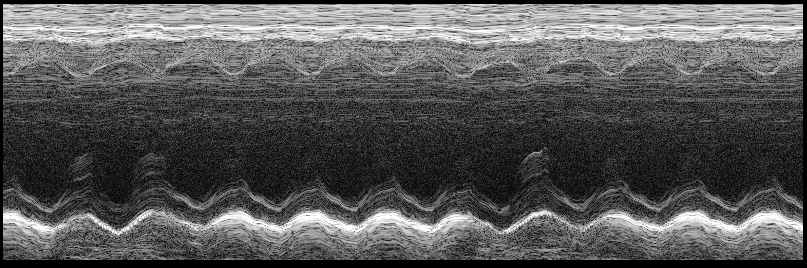

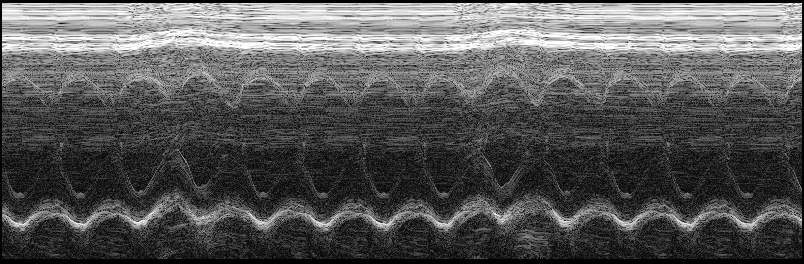

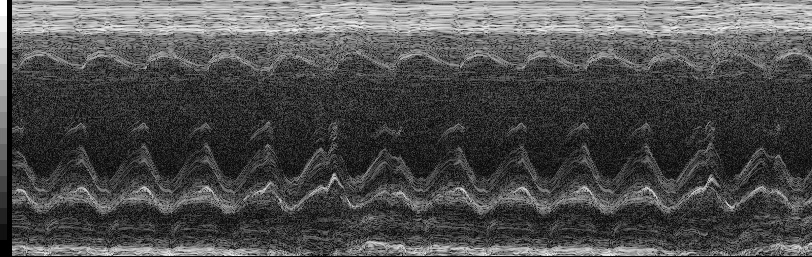


**SHAM+NS**

**IR+NS**

**IR+DLA**

**SHAM+DLA**

LVIDd

LVIDs

**Figure S4.** Representative echocardiograms of rat hearts from different groups for evaluation of the left ventricular structure and function. LVIDd, left ventricular internal diameter at end-diastole. LVIDs, left ventricular internal diameter at end-systole. n = 6.

Figure S5


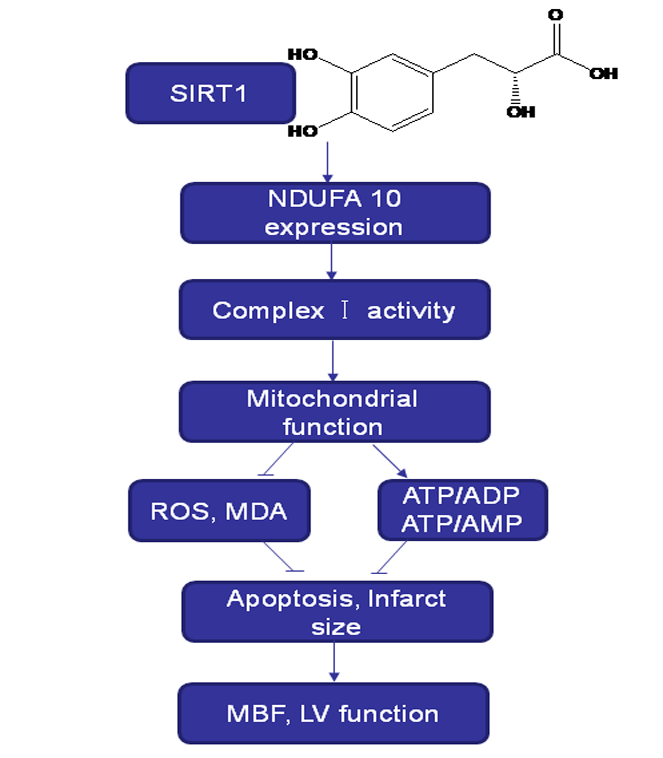


**Figure S5.** A diagram showing the pathway for DLA action. DLA binds to and actives SIRT1 depending on its phenolic hydroxyl, which promotes the expression of NDUFA10. Restored NDUFA10 expression improves Complex Ⅰ activity and mitochondrial function, which in turn reduces ROS and MDA production and increases the ratio of ATP/ADP and ATP/AMP, leading to better preserved MBF and LV function after I/R.
